# Dexmedetomidine attenuates haemorrhage-induced thalamic pain by inhibiting the TLR4/NF-κB/ERK1/2 pathway in mice

**DOI:** 10.1007/s10787-021-00877-w

**Published:** 2021-10-13

**Authors:** Tianfeng Huang, Yong Li, Wenqing Hu, Dapeng Yu, Ju Gao, Fan Yang, Yingying Xu, Zehua Wang, Liang Zong

**Affiliations:** 1 Department of Anesthesiology, Clinical Medical College of Yangzhou University, Northern Jiangsu People’s Hospital Affiliated with Yangzhou University, Yangzhou, Jiangsu People’s Republic of China; 2 Department of Gastrointestinal Surgery, Changzhi People’s Hospital, The Affiliated Hospital of Changzhi Medical College, Shanxi, No. 502 Changxing Middle Road, Luzhou District, Changzhi, 046000 People’s Republic of China; 3 Department of Central Laboratory, Changzhi People’s Hospital, Shanxi, The Affiliated Hospital of Changzhi Medical College, Changzhi, People’s Republic of China; 4 Department of General Surgery, Yizheng People’s Hospital, Clinical Medical College, Yangzhou University, No. 61 Dongyuan South Road, Yangzhou, 211400 Jiangsu People’s Republic of China; 5 Department of Anesthesiology, Heji Hospital Affiliated To Changzhi Medical College, No. 271 Taihang East Road, Changzhi, 046000 Shanxi People’s Republic of China

**Keywords:** Dexmedetomidine, Haemorrhage, Thalamic pain, Pathway, Inflammation

## Abstract

**Background:**

Thalamic pain, a neuropathic pain syndrome, frequently occurs after stroke. This research aimed to investigate the effect of dexmedetomidine (DEX) on thalamic pain.

**Methods:**

The cellular localization of the TLR4 protein was determined by immunostaining. The expression of Iba1, GFAP and protein associated with the TLR4/NF-κB/ERK1/2 pathway was measured by Western blotting. Continuous pain hypersensitivity was evaluated by behavioural tests. The results were analysed by one-way ANOVA, two-way ANOVA and Tukey’s post hoc test.

**Results:**

The results demonstrated that DEX obviously alleviated thalamic pain induced by haemorrhage on the ipsilateral side and delayed the development of pain hypersensitivity. Furthermore, the expression levels of Iba1, GFAP and proteins associated with the TLR4/NF-κB/ERK1/2 signalling pathway were greatly increased in mice with thalamic pain, but these effects were reversed by DEX.

**Conclusion:**

Our findings suggest that DEX alleviates the inflammatory response during thalamic pain through the TLR4/NF-κB/ERK1/2 signalling pathway and might be a potential therapeutic agent for thalamic pain.

**Supplementary Information:**

The online version contains supplementary material available at 10.1007/s10787-021-00877-w.

## Introduction

Currently, stroke, which has very high disability and mortality rates, is one of the most important diseases endangering human life and health worldwide. Central post-stroke pain (CPSP), a chronic neuropathic pain syndrome, is often induced by damage to and/or dysfunction of the central nervous system following stroke (Widar et al. 2002). CPSP occurs in approximately 1–14% of all stroke patients (Gritsch et al. 2016). CPSP is usually caused by damage to sensory pathways at various levels, such as the cerebral cortex, medulla oblongata and thalamus (Vartiainen et al. 2016). Patients with CPSP commonly experience long-term hyperalgesia, allodynia, spontaneous pain, and other sensory deficits, in some cases for the rest of their lives. Typical CPSP, also called thalamic pain syndrome or Dejerine–Roussy syndrome, refers to central pain caused by thalamic stroke. Thalamic haemorrhage is the main cause of CPSP (Klit et al. 2009; Shanthanna 2018). Haemorrhage in the ventral posterior lateral (VPL) nuclei, the ventral posterior medial (VPM) nuclei and the posterior (PO) nucleus of the thalamus is associated with a high incidence of CPSP. As the mechanism of CPSP has not yet been clarified and little attention is currently paid to central pain after stroke, the effect of drug treatment is not satisfactory, causing patients to suffer long-term pain that seriously affects quality of life and the ability to work and imposing a heavy burden on families and society.

The Toll-like receptor (TLR) family, a family of transmembrane pattern recognition receptors, mediates innate and adaptive immunity through recognized exogenous ligands, pathogen-associated molecular patterns and danger-associated molecular patterns (Akira et al. 2006). Toll-like receptor 4 (TLR4), the expression of which correlates with the prognosis of cerebral haemorrhage, plays a key role in the innate immune system and neuropathic pain (Zhang et al. 2018a, b; Piao et al. 2018). Inhibition of the TLR4/NF-κB signalling pathway alleviates cerebral damage and reduces brain water content after cerebral haemorrhage (Zhang et al. 2018a, b; Lan et al. 2017). Studies have also shown that inhibition of TLR4 can alleviate neuropathic pain associated with spinal cord injury (Chen et al. 2017) and peripheral neuropathic pain (such as post-operative pain and pain related to diabetic nerve injury) (Luo et al. 2018). However, whether the TLR4/NF-κB signalling pathway is related to thalamic pain is unknown.

Dexmedetomidine (DEX) is a novel selective α2 adrenergic receptor agonist. Excitation of α2 adrenergic receptors results in sedation, analgesia, and inhibition of sympathetic nerve activity through different signalling pathways (Mikami et al. 2017). A previous study suggested that DEX can exert analgesic effects against spinal cord injury-related neuropathic pain and peripheral neuropathic pain and that its analgesic effect increases with increasing dose. In addition, DEX can also reduce the dose of tramadol required to alleviate acute pain and neuropathic pain (Guneli et al. 2007). DEX can reduce sympathetic nerve excitability and ameliorate the neurological and histopathological alterations after cerebral ischaemic injury in rats. Interestingly, this effect can be reversed by α2 receptor antagonists (Sanders et al. 2005). Researchers have also found that DEX protects the brain to different degrees in animal models, such as a rabbit model of cerebral ischaemia/reperfusion injury (Maier et al. 1993) and a rat model of brain injury (Benggon et al. 2012). Degos et al. (2013) found that DEX can directly protect neuronal cells in studies of glutamate-mediated neuro-cytotoxicity. In addition, studies have revealed that DEX downregulates the expression of TLR4, a component of the central pro-inflammatory system, and upregulates the expression of the central anti-inflammatory system component nicotinic receptor a7nAChR, inhibiting the inflammatory response after injury (Rong et al. 2017). However, it is currently unknown whether DEX can alleviate CPSP caused by thalamic haemorrhage. In this study, a model of CPSP induced by thalamic haemorrhage was established, and the effect of DEX was observed.

## Materials and methods

### Animals

The study was approved by the Animal Care and Use Committee of the Medical College of Yangzhou University (Yangzhou, China) and conformed to the guidelines for animal care and use formulated by the Chinese government. Male CD1 mice aged approximately 7–8 weeks were purchased from the Comparative Medical Center of Yangzhou University, and housed in animal facilities. They were maintained on a 12-h light/dark cycle and provided free access to water and food pellets. All experiments were approved by the Animal Care and Use Committee of Yangzhou University and the International Association for Pain Research. To reduce variability in behavioural outcomes within and between individuals, the animals were trained in advance. The experimenter that performed the behavioural tests was blinded to the treatment conditions.

### Animal model of haemorrhage-induced thalamic pain

Anaesthetized animals were placed in a stereotaxic frame. Collagenase IV (Coll IV) (Sigma-Aldrich Co., St. Louis, MO; 0.01 U/10 nl) dissolved in saline solution was injected stereotaxically (Cai et al. 2018) into the VPM and VPL of the right thalamus of the mice (AP: − 0.82 to 2.30 mm, ML: − 1.30 to 1.95 mm from bregma and DV: − 3.01 to 4.25 mm below the dura into the right striatum). An equal volume of sterile saline was injected into the sham mice. After injected, the glass micropipette was kept in placed for 10 min to allow the Coll IV to completely disperse. Then, the glass micropipette was slowly removed. The wound was closed gently and cleaned with iodophor.

### Behavioural tests

Pain behaviour tests, including tests of mechanical, thermal and cold pain, were conducted.

First, the paw withdrawal latency in response to mechanical stimulation was measured (Li et al. 2017). The mice were placed in a plexiglass chamber with an elevated screen floor and allowed to adapt to the chamber for 30 min. The posterior limbs were stimulated for 1–2 s with 0.07 g and 0.4 g calibrated von Frey filaments (Stoelting Co.). The process was repeated 10 times at 5-min intervals. Quick withdrawal of the paw was considered a positive response. The paw withdrawal frequency was calculated as follows: (number of paw withdrawals/10 trials) × 100% = response frequency).

Next, an analgesia metre (model 336; IITC Inc. Life Science Instruments, Woodland Hills, CA) was used to measure the paw withdrawal latency as described previously (Li et al. 2015, 2017; Xu et al. 2014). A beam of light was delivered by the instrument and directed at the middle of the posterior paw of the mouse on the glass plate. Rapid withdrawal of the paw caused the beam of light to turn off. The duration for which the beam stimulated the paw was recorded as the paw withdrawal latency. Five trials were conducted for each side at an interval of 5 min. A cut-off time of 20 s was used to avoid tissue damage.

In addition, the paw withdrawal latency in response to a harmful cold stimulus (0 °C) was measured using a cold aluminium plate as described previously (Li et al. 2015, 2017; Xu et al. 2014). Briefly, mice were placed in a plexiglass chamber on a flat plate, the temperature of which was continuously monitored using a thermometer. The amount of time until the mice began jumping was recorded as the paw withdrawal latency. The experiment was carried out three times at an interval of 10 min.

After completion of the pain behaviour tests, motor function tests, including placement, grip and righting reflex tests, were performed as described previously (Li et al. 2017). For the placement reflex test, the hind limbs were placed slightly lower than the forelimbs, and the dorsal surface of the hind paws was brought into contact with the edge of the table. Whether the hind paws were reflexively placed on the table surface was recorded. For the grip reflex test, each mouse was placed on a wire grid, and whether the hind paws grasped the wire was recorded. For the righting reflex test, each mouse was placed on its back on a flat surface, and whether the mouse immediately returned to a normal upright position was recorded. Each test was repeated five times at a 5-min interval, and scores were recorded by calculating the number of normal responses.

### Western blot assay

Western blotting was performed as described in our previously published study (Huang et al. 2020). The protein concentration of each sample was measured using a BCA Protein Assay Kit, and then the samples were heated for 5 min at 99 °C. Next, 30 µg of protein was loaded in each well of a 10% polyacrylamide gel, and then the proteins on the gel were transferred to a polyvinylidene fluoride membrane. The PVDF membrane was incubated in 5% skimmed milk for 1 h, washed and incubated overnight at 4 °C with the following primary antibodies: rabbit anti-TLR4 (Cell Signaling Technology (CST); 1:1000), rabbit anti-phospho-p44/42 MAPK (Erk1/2) (CST; 1:2000), rabbit anti-p44/42 MAPK (Erk1/2) (CST; 1:2000), rabbit anti-phospho-NF-κB p65 (CST; 1:1000), rabbit anti-NF-κB p65 (CST; 1:1000), rabbit anti-Iba1 (FUJIFILM Wako Chemicals; 1:500), mouse anti-GFAP (CST; 1:2000) and rabbit anti-GAPDH (CST; 1:5000) followed by peroxidase-conjugated goat anti-mouse or anti-rabbit secondary antibodies (1:3000; Jackson ImmunoResearch) and a peroxidase-conjugated donkey anti-goat secondary antibody (1:2000; Jackson ImmunoResearch). Finally, the proteins were visualized using Western peroxide reagent and Clarity Western ECL Substrate (Bio-Rad) and the ChemiDoc XRS system (Bio-Rad) with Image Lab software. NIH ImageJ software was used to quantify the intensity of the bands by measuring the optical density.

### Immunofluorescence

The mice were anaesthetized with isoflurane and then perfused with 50–100 ml of physiological saline and 4% paraformaldehyde. The harvested brains were post-fixed in 4% paraformaldehyde overnight at 4 °C and dehydrated in 30% sucrose until they sank to the bottom of the container. Selected tissues were sliced into sections with a thickness of 30 µm. After blocking, these slices were incubated with primary antibodies, including a mouse anti-TLR4 antibody (Santa Cruz Biotechnology Inc; 1:50), a mixture of mouse anti-TLR (1:50) and rabbit anti-NeuN (a marker of neurons) (Invitrogen/Thermo Fisher Scientific; 1:500) antibodies, a rabbit anti-GFAP antibody (a marker for astrocytes) (Millipore Sigma, Burlington; 1:500), and a rabbit anti-Iba1 antibody (a marker of microglia) (FUJIFILM Wako Chemicals; 1:1000) overnight at 4 °C. Then, the sections were washed and incubated with secondary antibodies, including Cy2-conjugated donkey anti-mouse IgG Cy2 alone or a mixture of Cy2-conjugated donkey anti-mouse IgG and Cy3-conjugated goat anti-rabbit IgG, at room temperature for 1 h. All secondary antibodies were purchased from Jackson ImmunoResearch Labs (1:500). For control experiments, the primary antibody was replaced with normal mouse, rabbit, or goat serum. Finally, the sections were incubated with 4′,6-diamidino-2-phenylindole (DAPI) at room temperature for 15 min and mounted using VectaMount reagent (Vector Laboratories, Burlingame, CA). All images were captured with a Leica DMI4000 fluorescence microscope and DFC365FX camera. Single- and double-labelled cells were counted manually or using NIH ImageJ software.

### Statistical analysis

The animals were randomly assigned to various treatment groups. All the results are expressed as means ± SDs. The results were analysed by one-way ANOVA and two-way ANOVA. If ANOVA revealed a significant difference, a pairwise comparison of the mean values was performed using Tukey’s post hoc test (SigmaPlot 12.5, San Jose, CA). *P* < 0.05 was considered significant.

## Results

### The expression of TLR4 is increased after thalamic haemorrhage

**Fig. 1:**
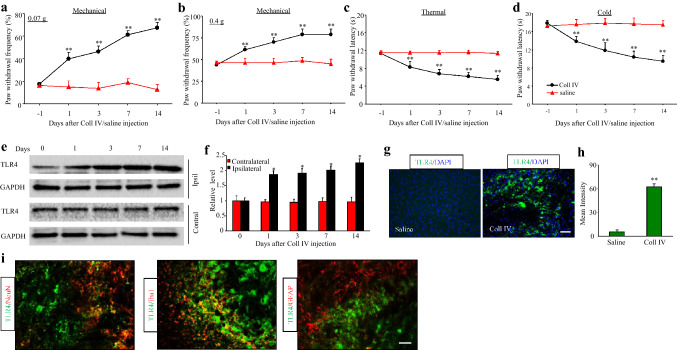
TLR4 expression was increased during thalamic pain. Microinjection of Coll IV into the ventral posterior medial nuclei and ventral posterior lateral nuclei led to increases in paw withdrawal frequencies in response to 0.07 g (**a**) and 0.4 g (**b**) von Frey filaments and decreases in the paw withdrawal latencies in response to stimulation of the ipsilateral side with heat (**c**) and cold (**d**) stimuli. *n* = 8. **P < 0.01 vs. the contralateral thalamus (**e**, **f**). Protein expression of TLR4 in the ipsilateral (Ipsi) and contralateral (Contra) thalamus on different days after microinjection of Coll IV. A representative Western blot (**e**). Statistical analysis of the densitometry data (**f**). *n* = 3. *P < 0.05 vs. the contralateral thalamus. Repeated paw withdrawal frequency and latency measurements and Western blot data were analysed by two-way ANOVA followed by Tukey’s post hoc test. (**g**, **h**) TLR4 immunofluorescence staining of the ipsilateral thalamus on day 7 after microinjection of Coll IV or saline. Representative immunofluorescence staining. Scale bar: 100 µm (**g**). Statistical analysis of the densitometry data (**h**). *n* = 3. **P < 0.01 vs. the saline-treated group by two-tailed unpaired Student’s *t* test. **i** TLR4 was co-localized with Iba1 and NeuN but not GFAP in the ipsilateral thalamus on day 7 after microinjection of Coll IV. *n* = 3. Scale bar: 100 µm

To evaluate pain hypersensitivity in the context of haemorrhage-induced thalamic pain, we measure the paw withdrawal frequencies of mice micro-injected with Coll IV into the VPM and VPL. Consistent with previous studies (Cai et al. 2018), the paw withdrawal frequencies in response to 0.07 g and 0.4 g von Frey filaments were markedly increased, proving that Coll IV induced long-lasting mechanical allodynia (Fig. 1a, b). The ipsilateral paw withdrawal latencies in response to heat (Fig. 1c) and cold (Fig. 1d) stimuli were significantly decreased, showing that heat and cold hyperalgesia occurred 1 day after microinjection of Coll IV and lasted for at least 14 days (Fig. 1a–d). However, the basal contralateral paw withdrawal responses were not significantly different (Supplementary Fig. 1a–c).

To confirm the function of TLR4, we evaluated the expression level of TLR4 in the thalami of mice with haemorrhage-induced thalamic pain after micro-injection of Coll IV (Cai et al. 2018). The expression level of TLR4 was persistently increased by 1.8-, 1.9-, 2.0- and 2.3-fold on days 1, 3, 7 and 14 after unilateral Coll IV microinjection, respectively, in the ipsilateral thalamus compared with the contralateral thalamus (Fig. 1e, f). Consistently, immunofluorescence staining with TLR4 antibodies showed that TLR4 immunoreactivity in the ipsilateral thalamus was weak in the saline-treated group (Fig. 1g); however, the density of TLR4-labelled cells was robustly increased by 12-fold on day 7 after microinjection of Coll IV (Fig. 1g, h). A double-labelling assay showed that the majority of the TLR4 signal in thalamic cells overlapped with the Iba1 signal (a marker of microglia) and that a small amount of the TLR4 signal in thalamic cells overlapped with the GFAP signal (a marker of astrocytes) and NeuN signal (a marker of neurons) on day 7 after Coll IV microinjection (Fig. 1i). This result indicated that TLR4 expression was increased mainly in microglia.

### Intraperitoneal (IP) administration of DEX attenuates the development of haemorrhage-induced thalamic pain

**Fig. 2:**
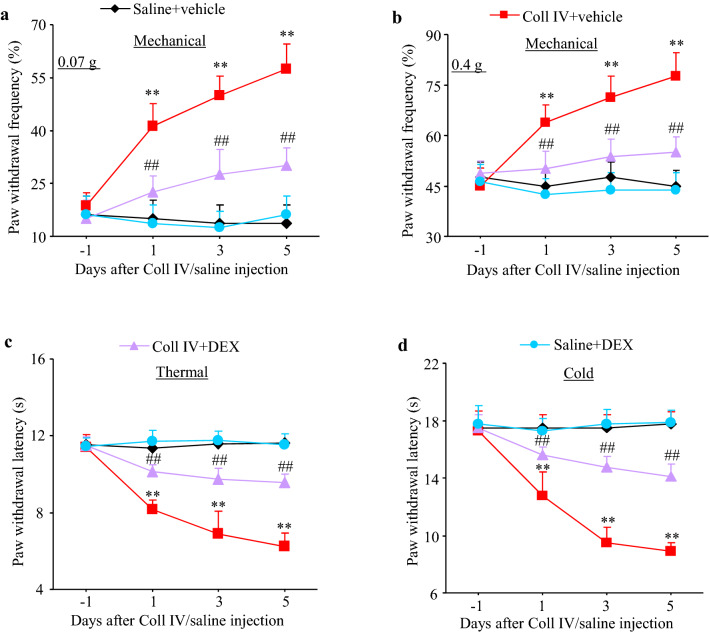
Effect of IP administration of DEX on thalamic pain development. DEX (40 μg/kg) or vehicle was given 30 min once daily before microinjection of Coll IV or saline. Effect of pre-administration of DEX or vehicle on paw withdrawal frequencies in response to 0.07 g (**a**) and 0.4 g (**b**) von Frey filaments and paw withdrawal latencies in response to heat (**c**) and cold (**d**) stimuli on different days after microinjection of Coll IV or saline. *n* = 8. Two-way repeated measures ANOVA followed by Tukey’s post hoc test. **P < 0.01 vs. day − 1. ^##^*P* < 0.01 vs. the Coll IV + vehicle-treated group

**Fig. 3:**
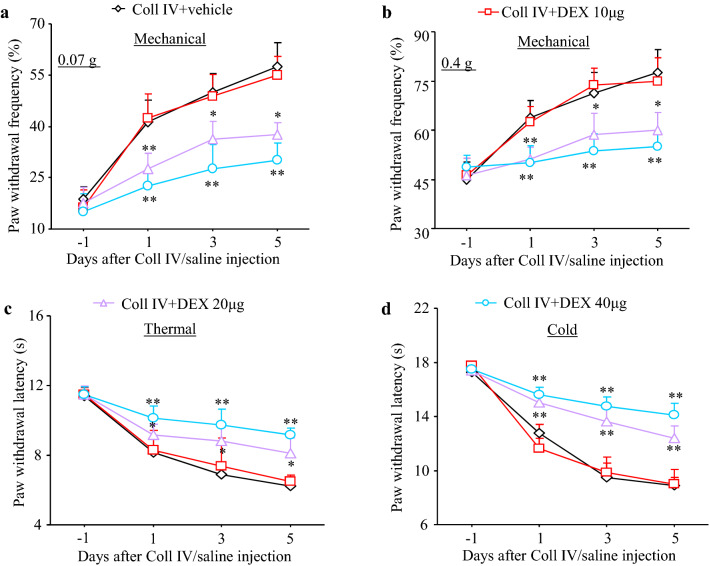
Dose-dependent effect of DEX on thalamic pain development. Effect of pre-administration of DEX [0 (vehicle), 10, 20, and 40 μg/kg] on paw withdrawal frequencies in response to 0.07 g (**a**) and 0.4 g (**b**) von Frey filaments and paw withdrawal latencies in response to heat (**c**) and cold (**d**) stimuli on days 1, 3 and 5 after microinjection of Coll IV. *n* = 8. Two-way repeated measures ANOVA followed by Tukey’s post hoc test. *P < 0.05 and **P < 0.01 vs. the Coll IV plus vehicle-treated group

We determined whether IP administration of DEX attenuated the development of haemorrhage-induced thalamic pain. DEX or vehicle was administered intraperitoneally 30 min before microinjection of Coll IV or saline and once daily for 5 days thereafter. Then, pain behaviour tests were performed on days 1, 3 and 5. As expected, mechanical allodynia, thermal hyperalgesia and cold hyperalgesia were observed on the contralateral (but not the ipsilateral) side in the vehicle plus Coll IV-treated group on days 1, 3 and 5 after Coll IV microinjection. These forms of pain hypersensitivity were significantly attenuated in Coll IV-microinjected mice administered 40 μg/kg DEX. (Fig. 2a–d). These effects were dose-dependent (Fig. 3a–d). DEX did not change the basal ipsilateral paw withdrawal frequency or latency in the DEX + Coll IV-treated group (Supplementary Fig. 2a–c) or the contralateral (Fig. 2a-d) or ipsilateral (Supplementary Fig. 2a–c) basal ipsilateral paw withdrawal frequency or latency in the DEX + saline-treated group. Vehicle did not influence the basal paw withdrawal frequency or latency in the saline-treated group (Fig. 2a–d; Supplementary Fig. 2a–c). After treatment, the locomotor activity of the mice recovered (Table 1).

**Table 1:** Locomotor tests

Treated group	Placing	Grasping	Ringhting
Saline + vehicle	5 (0)	5 (0)	5 (0)
Coll IV + vehicle	5 (0)	5 (0)	5 (0)
Coll IV + DEX	5 (0)	5 (0)	5 (0)
Saline + DEX	5 (0)	5 (0)	5 (0)

*N* = 8/group; 5 trials; mean (SD)

**Fig. 4:**
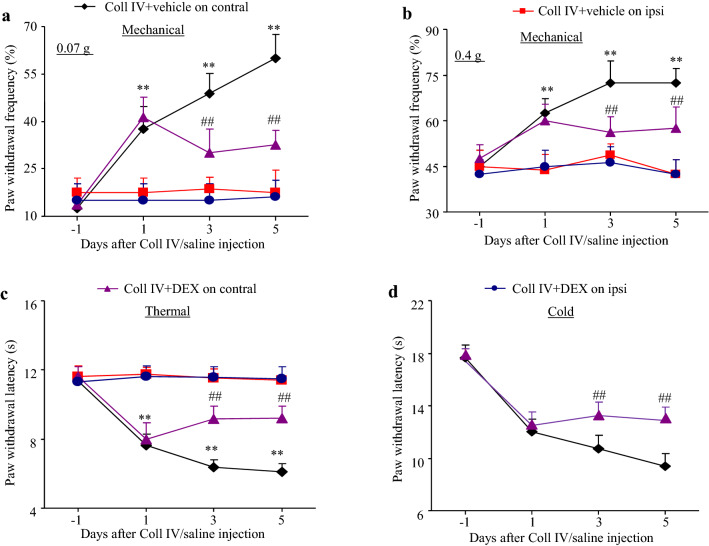
Effect of DEX on thalamic pain maintenance. DEX (40 μg/kg) or vehicle was given 1 day after Coll IV microinjection once daily for 5 days. Effect of DEX or vehicle on paw withdrawal frequencies in response to 0.07 g (**a**) and 0.4 g (**b**) von Frey filaments and paw withdrawal latencies in response to stimulation of the contralateral and ipsilateral side with heat (**c**) and cold (**d**) stimuli on days 3 and 5 after microinjection of Coll IV. *n* = 8. Two-way repeated measures ANOVA followed by Tukey’s post hoc test. **P < 0.01 vs. day − 1. ^##^*P* < 0.01 vs. the Coll IV + vehicle-treated group

The effect of DEX (40 μg/kg) on haemorrhage-induced thalamic pain maintenance was observed. After microinjection of Coll IV on day 1, robust mechanical allodynia and thermal heat and cold hyperalgesia developed (Fig. 4). Systemic administration of DEX (40 μg/kg) significantly ameliorated these forms of pain hypersensitivity on the ipsilateral side, but mechanical allodynia and thermal and cold hyperalgesia were observed in the vehicle-treated group on days 3 and 5 after microinjection of Coll IV (Fig. 4a–d). In addition, neither DEX nor vehicle altered the basal contralateral paw withdrawal responses (Fig. 4a–d).

TLR4 activates the NF-κB and ERK1/2 pathways in thalamic microglial cells, and DEX attenuates haemorrhage-induced thalamic pain by inhibiting the TLR4/NF-κB/ERK1/2 signalling pathway.

**Fig. 5:**
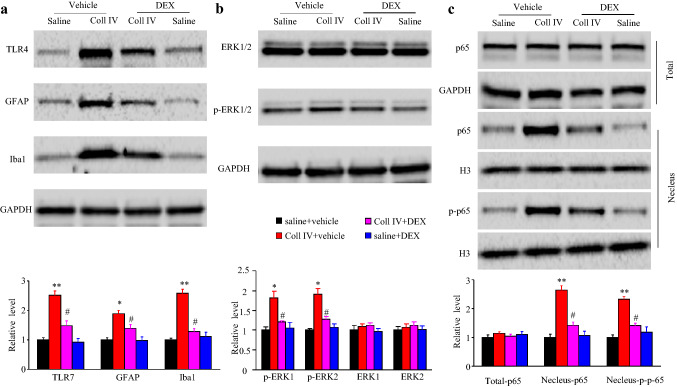
Effect of IP administration of DEX on activation of the NF-κB and ERK1/2 pathways in the thalamus. Expression of TLR4, Iba1 and GFAP in the cytoplasmic fraction (**a**), expression of p-ERK1/2 and ERK1/2 in the cytoplasmic fraction (**b**), expression of p-p65 and p-65 in the nuclear fraction and expression of p65 (total p65) in the total cellular fraction (**c**) of the ipsilateral thalamus in the different treatment groups on day 5 after microinjection of Coll IV or saline. *n* = 3. Two-way repeated measures ANOVA followed Tukey’s by post hoc test. *P < 0.05 and **P < 0.01 vs. the saline plus vehicle-treated group. ^#^*P* < 0.05 vs. the Coll IV plus vehicle-treated group

Finally, we explored the mechanism by which DEX attenuates haemorrhage-induced thalamic pain development. The expression level of TLR4 in the ipsilateral thalamus was increased by 2.5-fold in the vehicle + Coll IV-treated group compared with the vehicle + saline-treated group (Fig. 5a). However, the expression level of TLR4 was not increased in Coll IV + DEX-treated mice (Fig. 5a). Consistent with previous studies (Huang et al. 2020; Hanada et al. 2014), the expression levels of Iba1 and GFAP in the ipsilateral thalamus were remarkably increased in vehicle-treated mice on day 5 after microinjection of Coll IV, which demonstrated that microglia and astrocytes were activated (Fig. 5a). The increases in the expression levels of Iba1 and GFAP in Coll IV-treated mice were attenuated by systemic pre-treatment with DEX by IP injection (Fig. 5a). Furthermore, the NF-κB and ERK pathways were activated by Coll IV microinjection, as indicated by the increase in the expression of nuclear phosphorylated p65 (p-p65), nuclear p65 (He et al. 2020), phosphorylated ERK1/2 (p-ERK1/2) and ERK1/2 in the ipsilateral thalamus in vehicle-treated mice on day 5 after microinjection of Coll IV (Fig. 5b, c). However, systemic pre-treatment with DEX reversed these effects of Coll IV microinjection (Fig. 5b, c). However, the expression levels of total p65 and total ERK1/2 in the ipsilateral thalamus did not change (Fig. 5b, c).

## Discussion

Microinjection of Coll IV into the unilateral VPL and VPM of the murine thalamus leads to long-lasting pain hypersensitivity, including mechanical allodynia, thermal hyperalgesia and cold hyperalgesia on the contralateral side, mimicking thalamic pain caused by haemorrhagic stroke in humans (Kuan et al. 2015). However, the mechanisms underlying pain hypersensitivity development are still not clear. This study suggested that Coll IV increased the expression level of TLR4, which participates in the induction and maintenance of pain hypersensitivity, likely through the NF-κB and ERK1/2 signalling pathways in thalamic microglia. Furthermore, DEX administration decreased the expression levels of proteins related to the TLR4/NF-κB/ERK1/2 signalling pathway and reduced microglial activation. Our findings suggest that DEX may have therapeutic efficacy against haemorrhage-induced thalamic pain.

Current research on the pathogenesis of CPSP mainly focuses on neuro-inflammation, disinhibition and central sensitization. With extensive research on neuro-inflammation in the field of neurology, the role of neuro-inflammation in the mechanism of CPSP has become increasingly clear. The development of neuro-inflammation is thought to be related to microglia, astrocytes, and oligodendrocytes in the nervous system. Previous studies have revealed that in stroke, neuro-inflammation can cause secondary brain damage through activation of glial cells, which induces neuronal death, promotes the release of inflammatory cytokines, and leads to CPSP (Yang et al. 2017a, b). As innate macrophages and the smallest glial cells, microglia are distributed throughout the brain and account for approximately 5–10% of all glial cells. Microglia have become the focus of pain interventions. Recent research shows that glial cells and immune cells regulate the occurrence, development and maintenance of chronic pain through a variety of mechanisms, such as neuro-inflammation and glial crosstalk (Yang et al. 2014). Our study revealed that excessive activation of microglia after thalamic haemorrhage may lead to the occurrence of CPSP, which is consistent with the results of previous studies (Yang et al. 2017a, b; Lu et al. 2018).

TLR4 in the thalamus can be activated in response to haemorrhagic stroke, but its specific role in CPSP is unclear. Some studies have suggested that TLR4 plays a crucial role in other forms of neuropathic pain (Bettoni et al. 2008), including neuropathic pain caused by spinal cord injury (Ellis et al. 2016), hyperalgesia induced by opioids (Watkins et al. 2009), pain resulting from sciatic nerve compression (Luo et al. 2018), and postoperative pain (Kawano et al. 2016). After TLR4 is knocked out, pain is significantly relieved. TLR4 induces the development and maintenance of neuropathic pain, mainly by activating microglia and increasing the expression levels of the pro-inflammatory factors IL-1β and TNFα and the early-pain-mediator brain-derived nerve growth factor (BDNF) (Rodriguez-Yanez et al. 2012). Therefore, antagonizing the TLR4 signalling pathway might be a potential therapeutic strategy for thalamic pain. This study focused on whether TLR4-mediated neuro-inflammation is involved in central pain after stroke. The results of the study showed that the expression of TLR4, NF-κB and p-ERK1/2 at the edge of the thalamic lesion was increased in the CPSP group compared with the sham group, suggesting that the TLR4/NF-κB/ERK1/2 signalling pathway might be associated with pain.

DEX, a centrally selective α2 adrenergic receptor agonist, exerts good anxiolytic, sedative and analgesic effects (Cappuccio et al. 2018). DEX can produce analgesic effects in multiple sites, such as the rat brainstem, indicating that DEX can act on pain centres outside the spinal cord (Anger 2013). Kobayashi et al. (2011) found that intrathecal DEX administration has an anti-allodynic effect in a rat pain model. Liu et al. (2012) reported that systemic administration of DEX attenuates thermal and mechanical hyperalgesia in a partial sciatic nerve ligation (PSNL) model. In this study, pre-treatment with DEX significantly reduced pain hypersensitivity in mice with thalamic pain. The analgesic effect of DEX is dose-dependent. Moreover, our research also revealed that the administration of DEX in the maintenance phase of thalamic pain ameliorated pain hypersensitivity in mice with thalamic pain.

DEX can directly or indirectly act on neurons through microglia and astrocytes to exert its neuroprotective effect (Gupta et al. 2018). In our research, we found that DEX inhibited the activation of microglial cells. In addition, DEX pre-treatment can alleviate myocardial ischaemia/reperfusion injury in rats by inhibiting inflammatory processes through the TLR4/NF-κB signalling pathway (Yang et al. 2017a, b). In our study, the expression levels of proteins related to the TLR4/NF-κB/ERK1/2 signalling pathway were significantly increased, while DEX reversed these effects, indicating that DEX inhibits the TLR4/NF-κB/ERK1/2 pathway in a thalamic pain model.

## Conclusion

Our study revealed the effect of DEX on thalamic pain for the first time. The results showed that DEX alleviated the pain sensitivity in the context of thalamic pain. Additionally, DEX reduced the inflammatory response and the activation of glial cells, possibly through the TLR4/NF-κB/ERK1/2 signalling pathway.

## Supplementary Information

Below is the link to the electronic supplementary material.

Supplementary file1 Supplementary Fig. 1. Basal contralateral paw withdrawal response to 0.07 g (a) and 0.4 g (b) von Frey filaments and a decrease in the contralateral paw withdrawal latency in response to thermal (c) stimuli after Coll IV or saline injection. n = 8. Two-way repeated measures ANOVA followed by Tukey’s post hoc test (PPT 139 KB)

Supplementary file2 Supplementary Fig. 2. Basal contralateral paw withdrawal frequencies and latencies of the different groups in response to 0.07 g (a) and 0.4 g (b) von Frey filaments and heat (c) stimuli, respectively, after microinjection of Coll IV. Paw withdrawal frequencies in response to 0.07 g (d) and 0.4 g (e) von Frey filaments and the paw withdrawal latency in response to heat (f) stimuli after microinjection of Coll IV or DEX (0 (vehicle), 10, 20, or 40 μg/kg). n = 8. Two-way repeated measures ANOVA followed by Tukey’s post hoc test (PPT 202 KB)

## Data Availability

All data generated or analysed during this study are included in this published article.
