# Supplementary material for: Dexmedetomidine attenuates haemorrhage-induced thalamic pain by inhibiting the TLR4/NF-κB/ERK1/2 pathway in mice
Source: Inflammopharmacology. 2021 Oct 13;29(6):1751–60. doi: 10.1007/s10787-021-00877-w (PMC8643300; doi:10.1007/s10787-021-00877-w)
Supplement: Supplementary file 1 — Supplementary file1 Supplementary Fig. 1. Basal contralateral paw withdrawal response to 0.07 g (a) and 0.4 g (b) von Frey filaments and a decrease in the contralateral paw withdrawal latency in response to thermal (c) stimuli after Coll IV or saline injection. n = 8. Two-way repeated measures ANOVA followed by Tukey’s post hoc test (PPT 139 KB) [file 10787_2021_877_MOESM1_ESM.ppt]

## Slide 1
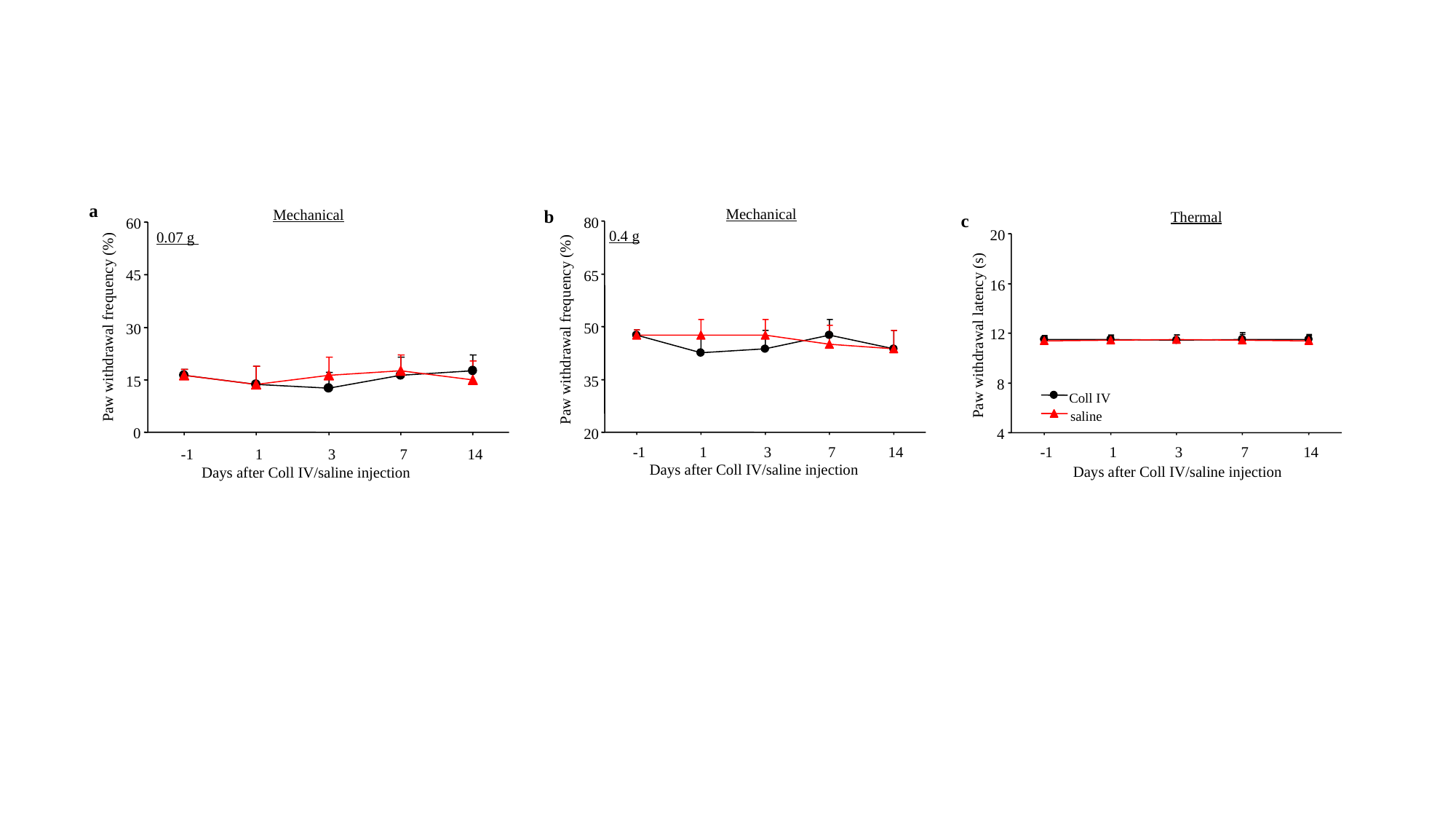

a
Mechanical
Mechanical
b
Thermal
c
80
60
20
0.4 g
0.07 g
45
65
16
Paw withdrawal frequency (%)
50
30
Paw withdrawal frequency (%)
12
Paw withdrawal latency (s)
15
35
8
Coll IV
saline
0
4
20
-1
1
3
7
14
-1
1
3
7
14
-1
1
3
7
14
Days after Coll IV/saline injection
Days after Coll IV/saline injection
Days after Coll IV/saline injection
