# Supplementary material for: Dexmedetomidine attenuates haemorrhage-induced thalamic pain by inhibiting the TLR4/NF-κB/ERK1/2 pathway in mice
Source: Inflammopharmacology. 2021 Oct 13;29(6):1751–60. doi: 10.1007/s10787-021-00877-w (PMC8643300; doi:10.1007/s10787-021-00877-w)
Supplement: Supplementary file 2 — Supplementary file2 Supplementary Fig. 2. Basal contralateral paw withdrawal frequencies and latencies of the different groups in response to 0.07 g (a) and 0.4 g (b) von Frey filaments and heat (c) stimuli, respectively, after microinjection of Coll IV. Paw withdrawal frequencies in response to 0.07 g (d) and 0.4 g (e) von Frey filaments and the paw withdrawal latency in response to heat (f) stimuli after microinjection of Coll IV or DEX (0 (vehicle), 10, 20, or 40 μg/kg). n = 8. Two-way repeated measures ANOVA followed by Tukey’s post hoc test (PPT 202 KB) [file 10787_2021_877_MOESM2_ESM.ppt]

## Slide 1
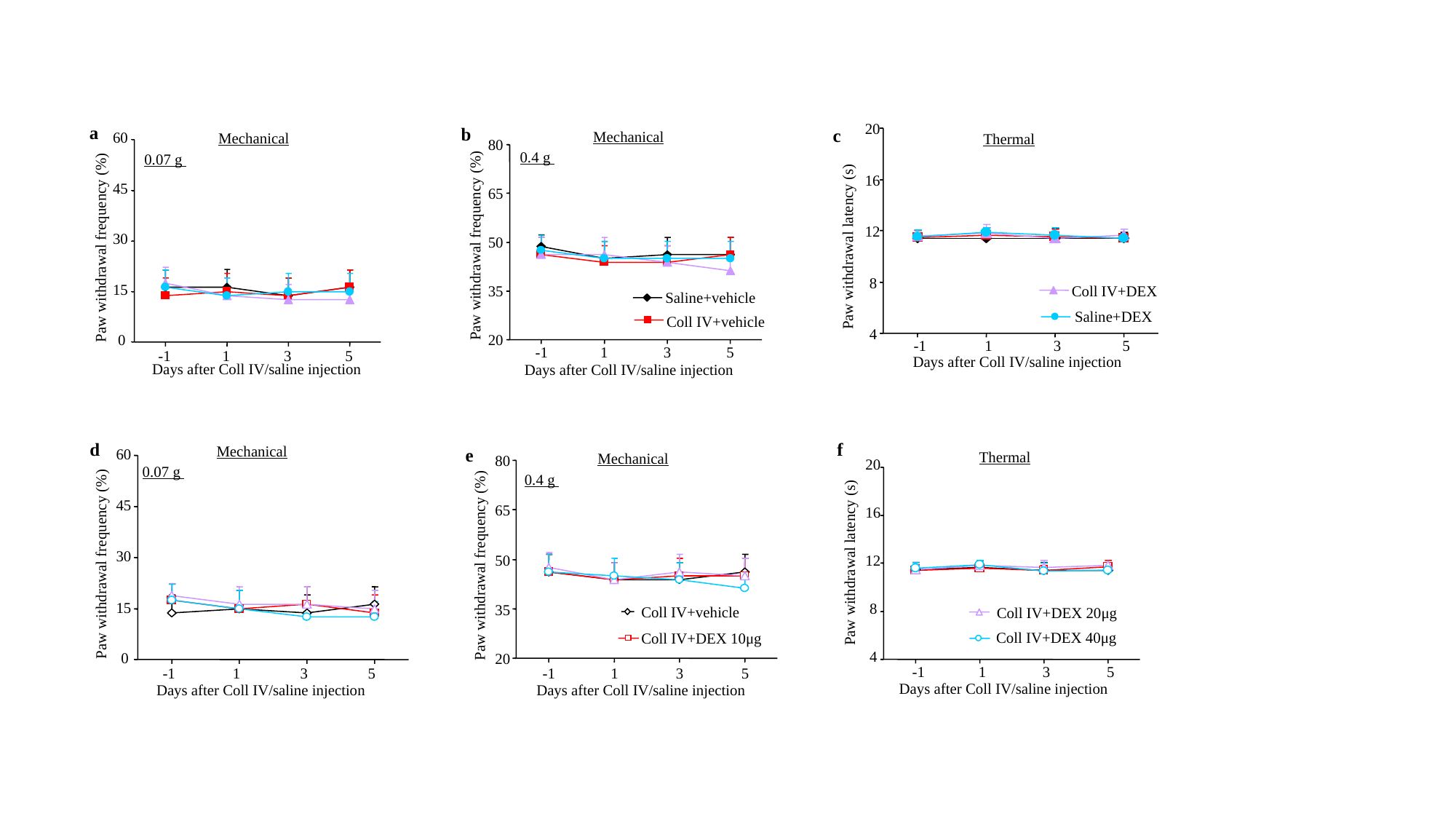

20
a
b
c
Mechanical
60
Mechanical
Thermal
80
0.4 g
0.07 g
16
45
65
12
30
50
Paw withdrawal frequency (%)
Paw withdrawal latency (s)
Paw withdrawal frequency (%)
8
15
Coll IV+DEX
35
Saline+vehicle
Saline+DEX
Coll IV+vehicle
4
20
0
-1
1
3
5
-1
1
3
5
-1
1
3
5
Days after Coll IV/saline injection
Days after Coll IV/saline injection
Days after Coll IV/saline injection
d
f
Mechanical
e
60
Thermal
Mechanical
80
20
0.07 g
0.4 g
45
65
16
30
12
50
Paw withdrawal latency (s)
Paw withdrawal frequency (%)
Paw withdrawal frequency (%)
15
8
35
Coll IV+vehicle
Coll IV+DEX 20μg
Coll IV+DEX 40μg
Coll IV+DEX 10μg
4
0
20
-1
1
3
5
-1
1
3
5
-1
1
3
5
Days after Coll IV/saline injection
Days after Coll IV/saline injection
Days after Coll IV/saline injection
